# Improvement of H_2_S Sensing Properties of SnO_2_-Based Thick Film Gas Sensors Promoted with MoO_3_ and NiO

**DOI:** 10.3390/s130303889

**Published:** 2013-03-19

**Authors:** Soo Chool Lee, Seong Yeol Kim, Byung Wook Hwang, Suk Yong Jung, Dhanusuraman Ragupathy, In Sung Son, Duk Dong Lee, Jae Chang Kim

**Affiliations:** 1 Department of Chemical Engineering, Kyungpook National University, Daegu 702-701, Korea; E-Mails: soochool@knu.ac.kr (S.C.L.); ksy256@naver.com (S.Y.K.); a048042@knu.ac.kr (B.W.H.); ojhyt@hanmail.net (S.Y.J.); 2 Centre for *Research* & Development, PRIST University, Puducherry 605 007, India; E-Mail: ragupathypdy@prist.ac.in; 3 HKC, Sucho-gu, Seoul 130-070, Korea; E-Mail: hkc1959@hkci.co.kr; 4 School of Electrical Engineering and Computer Science, Kyungpook National University, Daegu 702-701, Korea; E-Mail: ddlee@knu.ac.kr

**Keywords:** sensor, SnO_2_, pore size, H_2_S, MoO_3_, NiO

## Abstract

The effects of the SnO_2_ pore size and metal oxide promoters on the sensing properties of SnO_2_-based thick film gas sensors were investigated to improve the detection of very low H_2_S concentrations (<1 ppm). SnO_2_ sensors and SnO_2_-based thick-film gas sensors promoted with NiO, ZnO, MoO_3_, CuO or Fe_2_O_3_ were prepared, and their sensing properties were examined in a flow system. The SnO_2_ materials were prepared by calcining SnO_2_ at 600, 800, 1,000 and 1,200 °C to give materials identified as SnO_2_(600), SnO_2_(800), SnO_2_(1000), and SnO_2_(1200), respectively. The Sn(12)Mo5Ni3 sensor, which was prepared by physically mixing 5 wt% MoO_3_ (Mo5), 3 wt% NiO (Ni3) and SnO_2_(1200) with a large pore size of 312 nm, exhibited a high sensor response of approximately 75% for the detection of 1 ppm H_2_S at 350 °C with excellent recovery properties. Unlike the SnO_2_ sensors, its response was maintained during multiple cycles without deactivation. This was attributed to the promoter effect of MoO_3_. In particular, the Sn(12)Mo5Ni3 sensor developed in this study showed twice the response of the Sn(6)Mo5Ni3 sensor, which was prepared by SnO_2_(600) with the smaller pore size than SnO_2_(1200). The excellent sensor response and recovery properties of Sn(12)Mo5Ni3 are believed to be due to the combined promoter effects of MoO_3_ and NiO and the diffusion effect of H_2_S as a result of the large pore size of SnO_2_.

## 1. Introduction

Hydrogen sulfide (H_2_S) is an unwanted and toxic by-product of the coal, coal oil, and natural gas industries [1]. When hydrogen sulfide is emitted into the atmosphere, it is converted to SO_x_, which is a precursor to acid rain [2]. Accordingly, there is increasing demand for sensing devices that monitor low H_2_S concentrations. Well-known materials used to detect H_2_S include BaTiO_3_ [3], SnO_2_-Pd [4], Ag-SnO_2_ [5], SnO_2_-Al_2_O_3_ [6], SnO_2_-CuO [7–11], SnO_2_-CuO-SnO_2_ [12,13], SnO_2_-ZnO-CuO [14] and SiO_2_-doped Cu-Au-SnO_2_ [15]. Among the sensors described in the literature, CuO-modified thin-film or thick-film SnO_2_ sensors are promising for the sensitive and selective detection of H_2_S [1].

SnO_2_-based thick-film gas sensors have been used to detect toxic gases [16–28] on account of their high sensor response, simple design, low weight and low price. SnO_2_-based thick film gas sensors can achieve greater sensitivity to H_2_S through control of the particle size [17] and the addition of suitable promoters [13,14]. Wagh *et al.* reported that SnO_2_-ZnO-CuO thick-film sensors had significantly better response and recovery times than SnO_2_-ZnO or CuO doped SnO_2_ sensors [15]. Nevertheless, most studies on the sensing behavior of CuO-modified SnO_2_ thick-film gas sensors focused on concentrations of tens to hundreds of ppm. Until now, there have been very few studies of SnO_2_-based gas thick-film sensors for the detection of <1 ppm H_2_S.

In our previous papers, we described a SnO_2_-based thick-film gas sensor promoted with MoO_3_ and NiO, which was developed for the detection of dimethyl methylphosphonate (DMMP) and dichloromethane [26–28]. During the course of this earlier study, NiO and MoO_3_ promoters were found to play important roles in the sensor response and the recovery of the SnO_2_-based sensor, respectively, for the detection of toxic organic compounds containing P and Cl [26–28]. In the case of H_2_S detection, a SnO_2_-based thick-film sensor promoted with NiO and MoO_3_ showed improved recovery properties [2]. Nevertheless, the response of this sensor was decreased by promoting MoO_3_ despite the good recovery properties. Considering that the sensor response is an important factor in addition to the recovery properties, the improvement in the sensor response is necessary to develop a new SnO_2_-based thick-film gas sensor for the detection of <1 ppm H_2_S.

The aim of this study was to improve the response of a SnO_2_-based thick-film gas sensor promoted with NiO and MoO_3_ developed in a previous study for the detection of H_2_S at concentrations of <1 ppm. Accordingly, this study examined the effects of promoters and the textural properties of SnO_2_ on the sensing behaviors of SnO_2_-based thick-film sensors.

## 2. Experimental Section

### 2.1. Preparation of the Materials and Sensors

The SnO_2_ used as a source for the SnO_2_-based sensors was prepared from SnCl_4_ using a previously described ammonia-based precipitation method [2,26–28]. The products were calcined in a muffle furnace at various temperatures (600, 800, 1,000 or 1,200 °C). The SnO_2_-based materials were prepared by physically mixing two or three of the following promoters, NiO, ZnO, MoO_3_, CuO and Fe_2_O_3_, with SnO_2_. All products were calcined in a muffle furnace at 600 °C for 4 hours. The temperature ramp rate was 3 °C/min. The thick–film sensors were fabricated on an alumina substrate by screen-printing using a variety of physical mixtures, such as a SnO_2_-based powder and an organic binder (90% α-terpineol, Aldrich) [2,26–28]. The printed thick-film sensors were dried and calcined at 600 °C for 1 hour. This paper describes the sensors as SnO_2_(600) or Sn(6)Mo5Ni3, where (600) represents the calcination temperature, Sn(6) represents SnO_2_ calcined at 600 °C, Mn5 and Ni3 represent 5% MoO_3_ and 3% NiO, respectively, on a weight/weight basis.

### 2.2. Sensor Testing System

The sensing behaviors were examined in a flow system equipped with a 0.1 L chamber. The H_2_S gas was diluted with dry air to a concentration of <4.0 ppm. The total flow rate of the gas mixture was 400 mL/min. H_2_S gas was injected into chamber for 10 minutes. In the present study, the sensor response was defined using the following equation:

(1) $$\text{Sensor respons}(\%)=[({\text{R}}_{\text{a}}-{\text{R}}_{\text{g}})/{\text{R}}_{\text{a}}]\times 100$$

where R_a_ and R_g_ are the electric resistance in air and test gas, respectively. The sensor recovery was defined by the following equation:

(2) $$\text{Recovery}(\%)=[({\text{S}}_{\text{m}}-{\text{S}}_{\text{r}})/{\text{S}}_{\text{m}}]\times 100$$

where S_m_ and S_r_ represent the maximum sensor response over a period of 10 minutes and the minimum sensor response in air, respectively.

### 2.3. Characterization of Materials

The crystalline phases in the materials were identified by power X-ray diffraction (XRD; Philips, X′PERT) using Cu Kα radiation. The morphology of the SnO_2_ powder was observed by transmission electron microscopy (TEM; Hitachi, H-7100), and the textural properties of the materials were examined using an Hg porosimetry (Micromeritics, AutoPore IV 9500).

## 3. Results and Discussion

### 3.1. Effects of SnO_2_ Pore Size on Sensor Properties

**Figure 1.**
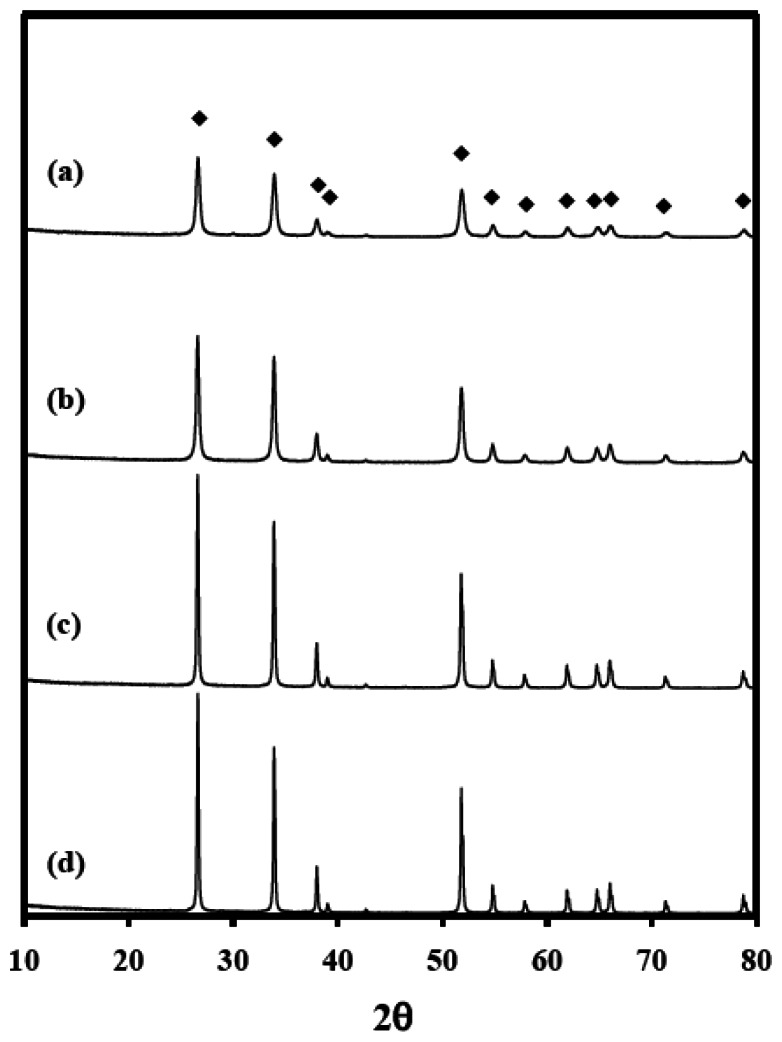
XRD patterns of SnO_2_ materials calcined at (**a**) 600; (**b**) 800; (**c**) 1,000; and (**d**) 1,200 °C; (◆) SnO_2_.

To examine the effects of the textural properties of SnO_2_ on the sensing properties, the SnO_2_ materials were prepared by calcining SnO_2_ at temperatures of 600, 800, 1000, and 1,200 °C [affording materials identified as SnO_2_(600), SnO_2_(800), SnO_2_(1000), and SnO_2_(1200), respectively]. Figure 1 shows XRD patterns of the SnO_2_ materials.

Diffraction peaks were observed at 26.6, 33.8, 37.9, 51.8, 54.8, 61.9, 64.7, 65.9 and 71.3° 2θ, and the intensities of these diffraction peaks increased with increasing temperature, indicating an increase in the crystallite size and the crystallinity [29,30], but the structures of SnO_2_ were retained. To confirm these results, the sizes of the SnO_2_ crystallites were calculated from the XRD patterns using Scherrer's equation Equation (3). As expected, the crystallite size of SnO_2_ increased from 19 to 54 nm with increasing calcination temperature (Table 1).

(3) $$\text{t}=(\text{K}\cdot \lambda )/({\text{W}}_{\text{size}}\cdot \cos\theta )=(0.9\cdot \lambda )(\text{FWHM}\cdot \cos\theta )$$

In a separate experiment, TEM images of these SnO_2_ materials were investigated. Table 1 lists the crystallite sizes obtained from TEM images, which concur with those determined by XRD. Table 2 lists the textural properties of SnO_2_ materials, as determined by Hg porosimetry. The surface areas decreased with increasing calcination temperature, whereas the average pore diameters increased, presumably because the pore diameter is dependent on the crystallite size.

**Figure 2.**
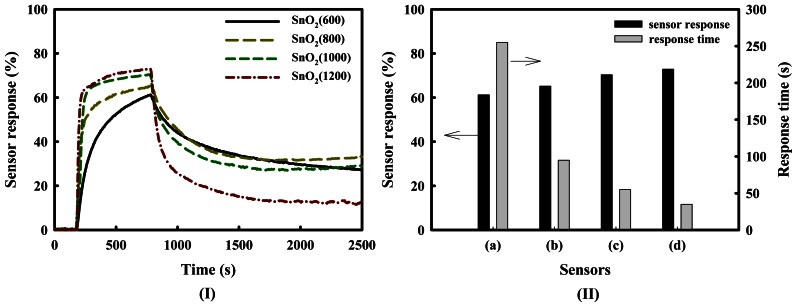
(**I**) Response curves, (**II**) responses, and (II) 80% response times of SnO_2_-based gas sensors, such as (a) SnO_2_(600); (b) SnO_2_(800); (c) SnO_2_(1000); and (d) SnO_2_(1200) at a H_2_S concentration of 1.0 ppm at 350 °C.

Figure 2 shows the response curves, responses and 80% response times of SnO_2_(600), SnO_2_(800), SnO_2_(1000) and SnO_2_(1200) gas sensors at a H_2_S concentration of 1.0 ppm at 350 °C. The responses of the SnO_2_-based sensors increased in the following order: SnO_2_(600) < SnO_2_(800) < SnO_2_(1000) < SnO_2_(1200). The response time of the SnO_2_(1200) sensor was much shorter than that of the SnO_2_(600) sensor, even though sensor recovery was incomplete in air. These results mean that the response time decreases with increasing pore diameter, as shown in Table 1 and Figure 2(II), and the sensor response increases. However, the important point to note is the incomplete recovery of the sensors after the detection of H_2_S, despite the high sensor response. It is thought that this result is because sulfur compounds are adsorbed on the sensor's surface, and that they progressively pollute the surface of tin dioxide.

**Figure 3.**
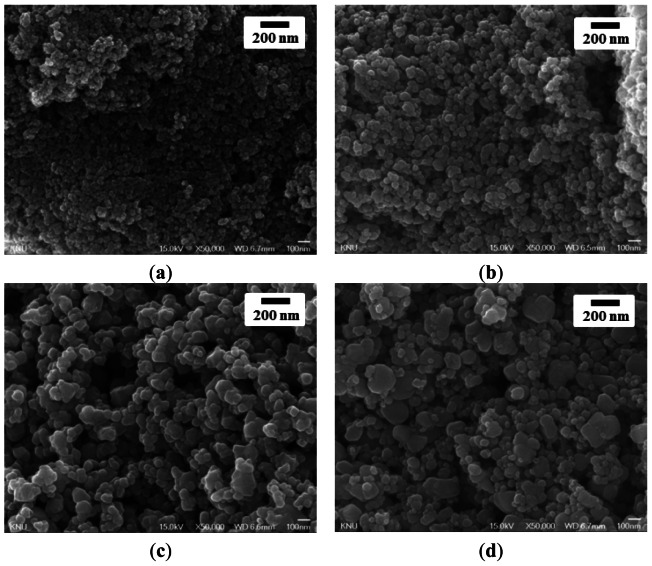
SEM images of the SnO_2_ thick-film sensors: (**a**) SnO_2_(600); (**b**) SnO_2_(800); (**c**) SnO_2_(1000); and (**d**) SnO_2_(1200).

Figure 3 shows SEM images of the surfaces of the SnO_2_(600), SnO_2_(800), SnO_2_(1000) and SnO_2_(1200) thick-film sensors. The particle size of SnO_2_ increased with increasing calcination temperature in the following order: SnO_2_(600) < SnO_2_(800) < SnO_2_(1000) < SnO_2_(1200). Liu *et al.* reported that the sensor sample based on SnO_2_ nanocrystals produced by the gel combustion method had higher response and shorter response times, which might be due to the more porous nano-crystallinity (∼50 nm in size) than the sample prepared from hydrothermal-synthesized SnO_2_ nanocrystals, where smaller SnO_2_ nanocrystals (∼12–13 nm) are densely packed and agglomerate into large entities (secondary particles), approximately 2–3 μm in size [17]. However, in this study, particle size after screen-printing, as well as average pore diameter of SnO_2_, is directly related to the crystallite size (Table 1) and the crystalinity of SnO_2_, which is in contrast to Liu *et al.*'s results. This result is because the crystallite size and the crystalinity of SnO_2_ was controlled by calcining the SnO_2_ material, which was prepared by precipitation, at various temperatures (600, 800, 1,000 and 1,200 °C), and the SnO_2_ thick–film sensors were fabricated on an alumina substrate by screen-printing using these calcined materials. From these results, it is clear that the sensor response and response time for the detection of H_2_S gas are directly affected by the SnO_2_ pore diameter rather than to the surface area due to the diffusion of H_2_S gas. In particular, the important point to note is that the response time of the SnO_2_ sensor, as well as sensor response for the detection of H_2_S, can be enhanced by increasing the crystallite size of SnO_2_ by calcination.

### 3.2. Promoter Effects on Sensor Response and Recovery

**Figure 4.**
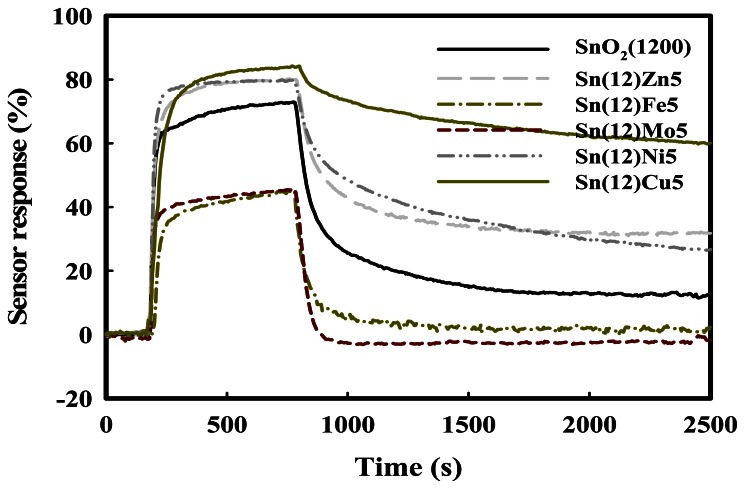
Response curves of the SnO_2_-based gas sensors promoted with various metal oxides at a H_2_S concentration of 1.0 ppm at 350 °C.

**Figure 5.**
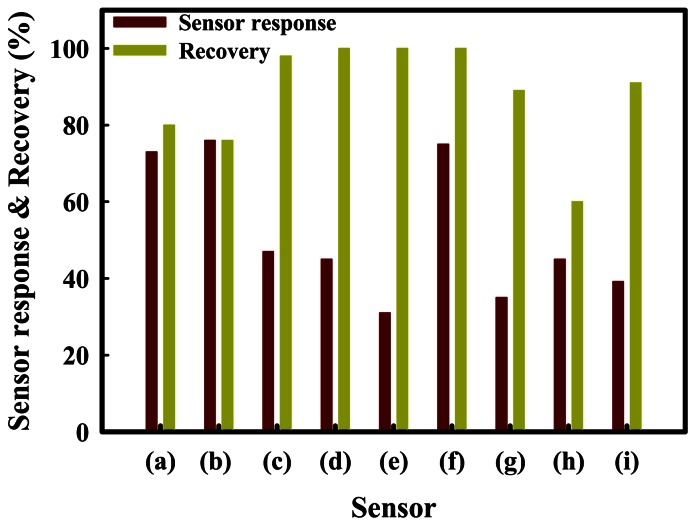
Responses and recoveries of the SnO_2_-based gas sensors promoted with various amounts of metal oxides at a H_2_S concentration of 1.0 ppm at 350 °C; (a) SnO_2_(1200); (b) Sn(12)Mo1; (c) Sn(12)Mo3; (d) Sn(12)Mo5; (e) Sn(12)Mo5Ni1; (f) Sn(12)Mo5Ni3; (g) Sn(12)Mo5Ni5; (h) Sn(12)Mo5Zn3; and (i) Sn(6)Mo5Ni3.

The sensor recovery properties are an important consideration for sensors designed for H_2_S. To improve the sensor recovery, SnO_2_(1200)-based sensors were prepared by physically mixing with ZnO, Fe_2_O_3_, MoO_3_, NiO, or CuO promoters, which are referred to as Sn(12)Zn, Sn(12)Fe5, Sn(12)Mo5, Sn(12)Ni5, and Sn(12)Cu5, respectively. The effects of the promoters on the sensor recovery properties were investigated at a H_2_S concentration of 1.0 ppm at 350 °C. The results obtained are summarized in Figure 4. The Sn(12)Zn5, Sn(12)Ni5 and Sn(12)Cu5 sensors showed a slight increase in the sensor response compared to the SnO_2_ sensor, but their recoveries were incomplete at 350 °C. On the other hand, Sn(12)Fe5 and Sn(12)Mo5 showed complete recovery, but exhibited much lower responses than the SnO_2_(1200) sensor. In particular, the Sn(12)Mo5 sensor showed a faster recovery time than the Sn(12)Fe5 sensor, and a response that was approximately 42% higher than that of the SnO_2_(600)-based sensor containing 5 wt% MoO_3_ [Sn(6)Mo5]. The reason for excellent recovery properties of the Fe5 and Mo5 sensors is not clear yet, but it is thought that Fe_2_O_3_ and MoO_3_ promoters added to SnO_2_ play an important role in the desorption of sulfur compounds. To identify the effects of the promoters on the sensor response and recovery, the SnO_2_-based sensors promoted with various amounts of metal oxides (MoO_3_, NiO_3_, and ZnO) were examined at 1 ppm H_2_S and 350 °C. These results are shown in Figure 5. As shown by Figures 5(b,c) and (d), the Sn(12)Mo5 sensor achieved a recovery of 100%, even though the sensor response was decreased by the MoO_3_ promoter. Previous studies found that NiO plays an important role in enhancing the sensor response of the SnO_2_-based sensor promoted with MoO_3_ for the detection of dimethyl methylphosphonate (DMMP) and dichloromethane [23,24]. In the present study, the sensor response for the detection of H_2_S was increased by NiO (Figure 5). As expected, the Sn(12)Mo5Ni3 sensor, which was promoted with both MoO_3_ and NiO, showed a sharp increase in the sensor response and maintained the sensor recovery properties (Figure 5(f)). In particular, the Sn(12)Mo5Ni3 sensor exhibited much higher sensor response and recovery than the Sn(6)Mo5Ni3 sensor [2] (39.2% and 91%, respectively). These results are attributed to diffusion effects caused by the larger pore size of SnO_2_ and the promoter effects of NiO and MoO_3_. However, further study is required to verify the sensing mechanisms and the roles of NiO, ZnO, CuO, MoO_3_, and Fe_2_O_3_ promoters in the sensor response and recovery properties.

**Figure 6.**
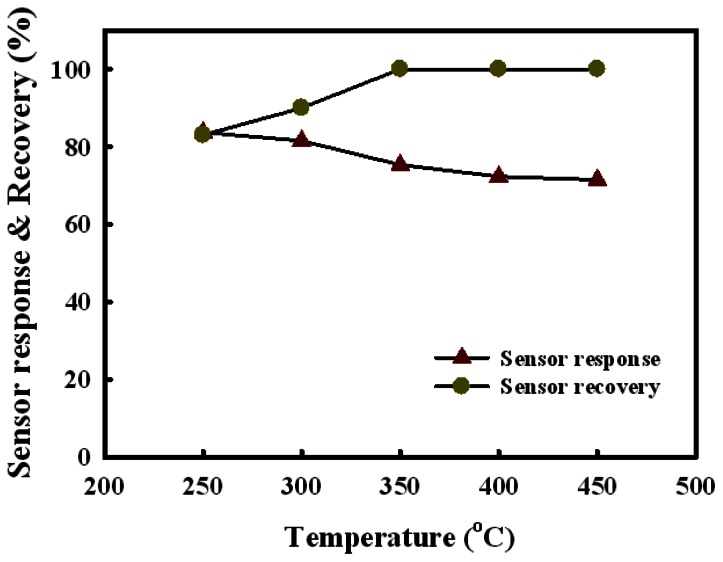
Responses and recovery of the Sn(12)Mo5Ni3 sensor as a function of the detection temperature at a H_2_S concentration of 1.0 ppm.

Figure 6 shows the response and recovery of the Sn(12)Mo5Ni3 sensor as a function of temperature at a H_2_S concentration of 1.0 ppm. The sensor response decreased slightly with increasing detection temperature, whereas the sensor recovery increased between 250 °C and 350 °C. Considering the sensor response and recovery, the optimum temperature for the detection of H_2_S was 350 °C.

**Figure 7.**
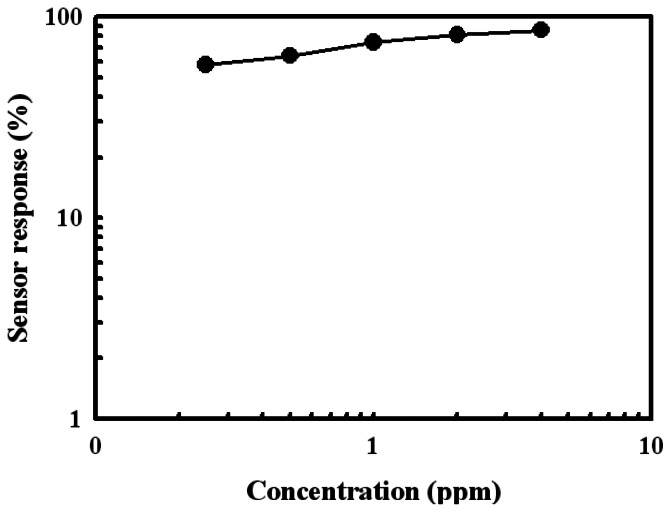
Response of the Sn(12)Mo5Ni3 sensor as a function of the H_2_S concentration.

Figure 7 shows the response of the Sn(12)Mo5Ni3 sensor at concentrations between 0.25 ppm and 4 ppm at 350 °C. The response of this sensor increased almost linearly between 0.25 ppm and 4 ppm. The Sn(12)Mo5Ni3 sensor had a high sensor response of approximately 59% at low H_2_S concentrations of 0.25 ppm.

**Figure 8.**
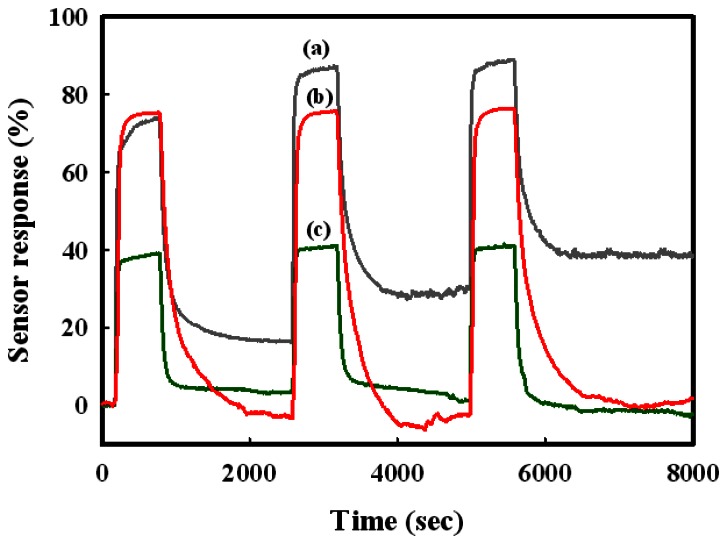
Repeatabilities of the (a) SnO_2_(1200); (b) Sn(12)Mo5Ni3; and (c) Sn(6)MoNi3 sensors.

Figure 8 shows the repeatabilities of the SnO_2_(1200), Sn(12)Mo5Ni3, and Sn(6)Mo5Ni3 sensors at a H_2_S concentration of 1 ppm and 350 °C. The response of the SnO_2_(1200) sensor decreased gradually over multiple detection and recovery tests. On the other hand, the Sn(12)Mo5Ni3 sensor maintained its response over multiple tests without deactivation. The response of the Sn(12)Mo5Ni3 sensor was approximately double that of the Sn(6)Mo5Ni3 sensor.

**Figure 9.**
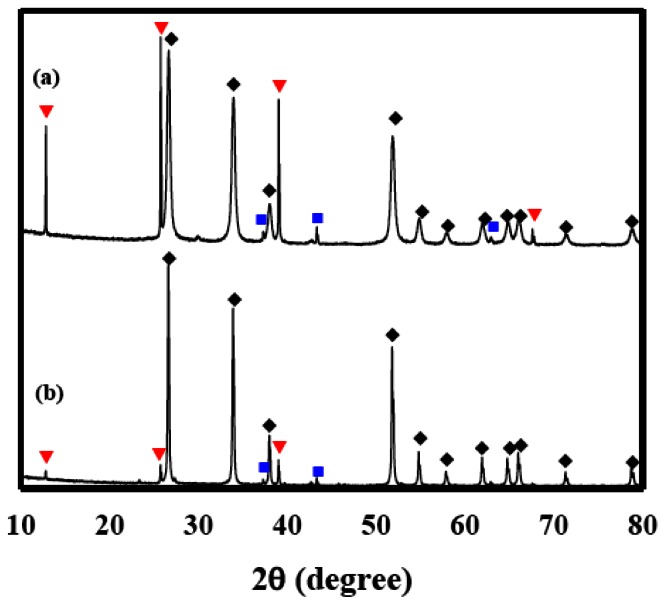
XRD patterns of the (**a**) Sn(6)Mo5Ni3 and (**b**) Sn(12)Mo5Ni3 materials; (◆); SnO_2_, (nO MoO_3_, and (nd NiO.

**Figure 10.**
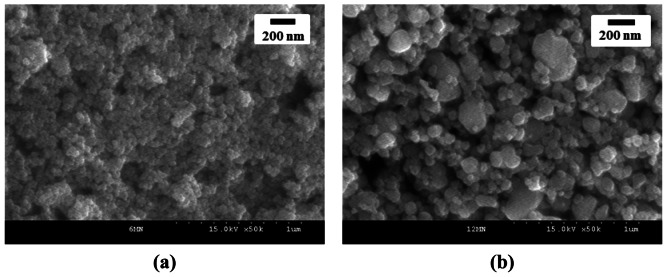
SEM images of the (**a**) Sn(6)Mo5Ni3 and (**b**) Sn(12)Mo5Ni3 sensors.

Figure 9 shows XRD patterns of Sn(6)Mo5Ni3 and Sn(12)Mo5Ni3 materials. Their XRD patterns showed MoO_3_ (JCPDS No. 89-7112), NiO (JCPDS No. 89-7390) and SnO_2_ (JCPDS No. 88-0287) phases. The diffraction peaks of these two materials were similar, as shown in Figure 9(a,b), which suggests that the observed enhancement in sensor response cannot be explained by structural differences alone. SEM images of the Sn(6)Mo5Ni3 and Sn(12)Mo5Ni3 sensors were observed at ×50 K and these results were shown in Figure 10. There is no change in the morphologies of those sensors as compared with the SnO_2_(600) and SnO_2_(1200) sensors. Table 3 lists the textural properties of the Sn(6)Mo5Ni3 and Sn(12)Mo5Ni3 materials determined by Hg porosimetry. The mean pore diameter of Sn(12)Mo5Ni3 was approximately double that of Sn(6)Mo5Ni3 (Table 3). This means that the pore size of SnO_2_ and the promoter play important roles in the sensor response to H_2_S. Based on these results, we believe that it is possible to prepare an excellent SnO_2_-based sensor for the detection of H_2_S at concentrations of < 1 ppm with a high sensor response and excellent recovery properties using SnO_2_ with a large pore size, in conjunction with NiO and MoO_3_ promoters.

## 4. Conclusions

A new large pore size SnO_2_-based thick-film gas sensor promoted with MoO_3_ and NiO [Sn(12)Mo5Ni3] was developed for the detection of H_2_S at 350 °C. This sensor exhibited 100% recovery at 350 °C and a maximum sensor response of 75%, and maintained a sensor response of 75% over many operating cycles without deactivation at a H_2_S concentration of 1 ppm and 350 °C. In addition, its response increased almost linearly between 0.25 and 1 ppm. Furthermore, the sensor exhibited a high response (59%) at a H_2_S concentration of only 0.25 ppm. In particular, the Sn(12)Mo5Ni3 sensor exhibited double the response of the corresponding Sn(6)Mo5Ni3 sensor, which was prepared by adding MoO_3_ and NiO to SnO_2_ calcined at 600 °C. These results are explained by the promoter effects of MoO_3_ and NiO, and the diffusion effects associated with a large SnO_2_ pore size.

## Figures and Tables

**Table 1.** Crystallite sizes calculated using XRD and TEM data.

**SnO_2_materials**	**XRD**				**TEM**

	**Wave Length** **(nm)**	**2θ** **(°)**	**FWHM** **(cm^3^/g)**	**Crystallite Size** **(nm)**	**Crystallite Size** **(nm)**
SnO_2_(600)	0.154	26.611	0.4095	19	10–20
SnO_2_(800)	0.154	26.581	0.3104	26	25–30
SnO_2_(1,000)	0.154	26.585	0.1690	47	40–50
SnO_2_(1,200)	0.154	26.604	0.1488	54	50–70

**Table 2.** Textural properties of the SnO_2_ materials produced by Hg porosimetry.

**SnO_2_Materials**	**Surface Area (m^2^/g)**	**Pore Volume (cm^3^/g)**	**Average Pore Diameter (nm)**
SnO_2_(600)	24.8	0.4918	79
SnO_2_(800)	16.4	0.5047	122
SnO_2_(1000)	9.4	0.5226	222
SnO_2_(1200)	8.0	0.6263	312

**Table 3.** Textural properties of the Sn(6)Mo5Ni3 and Sn(12)Mo5Ni3 by Hg porosimetry.

**SnO_2_Materials**	**Surface Area (m^2^/g)**	**Pore Volume (cm^3^/g)**	**Average Pore Diameter (nm)**
Sn(6)Mo5Ni3	11.8	0.5197	175.6
Sn(12)Mo5Ni3	4.7	0.4019	338.6
